# Weak measurements and quantum-to-classical transitions in free electron–photon interactions

**DOI:** 10.1038/s41377-023-01292-2

**Published:** 2023-11-08

**Authors:** Yiming Pan, Eliahu Cohen, Ebrahim Karimi, Avraham Gover, Norbert Schönenberger, Tomáš Chlouba, Kangpeng Wang, Saar Nehemia, Peter Hommelhoff, Ido Kaminer, Yakir Aharonov

**Affiliations:** 1 School of Physical Science and Technology and Center for Transformative Science, ShanghaiTech University, Shanghai, 200031 China; 2 Department of Electrical Engineering, Technion, Haifa, 3200003 Israel; 3 Department of Physics of Complex Systems, Weizmann Institute of Science, Rehovot, 7610001 Israel; 4 Faculty of Engineering and the Institute of Nanotechnology and Advanced Materials, Bar Ilan University, Ramat Gan, 5290002 Israel; 5 Department of Physics, University of Ottawa, Ottawa, Ontario K1N 6N5 Canada; 6 Department of Electrical Engineering Physical Electronics, Center for Laser–Matter Interaction (LMI), Tel Aviv University, Ramat Aviv, 6997801 Israel; 7 Department of Physics, Friedrich-Alexander Universität Erlangen-Nürnberg (FAU), Staudtstraße 1, 91058 Erlangen, Germany; 8 School of Physics and Astronomy, Tel Aviv University, Ramat Aviv, 6997801 Israel; 9 Institute for Quantum Studies, Chapman University, Orange, CA 92866 USA

**Keywords:** Nanophotonics and plasmonics, Ultrafast photonics, Quantum optics

## Abstract

How does the quantum-to-classical transition of measurement occur? This question is vital for both foundations and applications of quantum mechanics. Here, we develop a new measurement-based framework for characterizing the classical and quantum free electron–photon interactions and then experimentally test it. We first analyze the transition from projective to weak measurement in generic light–matter interactions and show that any classical electron-laser-beam interaction can be represented as an outcome of weak measurement. In particular, the appearance of classical point-particle acceleration is an example of an amplified weak value resulting from weak measurement. A universal factor, $\exp \left(-{\Gamma }^{2}/2\right)$, quantifies the measurement regimes and their transition from quantum to classical, where $\Gamma$ corresponds to the ratio between the electron wavepacket size and the optical wavelength. This measurement-based formulation is experimentally verified in both limits of photon-induced near-field electron microscopy and the classical acceleration regime using a DLA. Our results shed new light on the transition from quantum to classical electrodynamics, enabling us to employ the essence of the wave-particle duality of both light and electrons in quantum measurement for exploring and applying many quantum and classical light–matter interactions.

## Abstract (web summary)

We explore the transition from quantum to classical electrodynamics in light–matter interactions using a novel measurement-based approach. Our findings demonstrate that classical electron–photon couplings can be explained through weak measurement.

## Graphical abstract

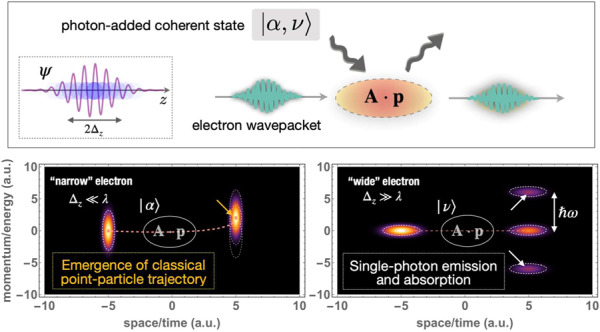

## Introduction

Measurement lies at the heart of quantum mechanics and allows one to probe a quantum system of interest through a measuring pointer (an apparatus) coupled to the system’s observables. The interaction between the system and pointer is later classically amplified for the outcome to be seen macroscopically. However, in the context of light–matter interactions, sometimes either the measured system or measuring pointer (or both) can be well treated with classical means, i.e., without invoking quantum formalism. These interactions are usually modelled by classical or quantum electrodynamics, with a wealth of widely explored effects and both theoretical and experimental schemes such as photon-induced near-field electron microscopy (PINEM)^1–5^ or dielectric laser accelerator (DLA)^6–8^. All these inspire our current exploration. We show that there is a continuous transition from quantum to classical interactions between electrons and photons, which can be illustrated when examining several prototypical scenarios of the measurement in experimental settings. Previously, it was demonstrated that post-selection and weak measurements can provide insights regarding the boundary between classical and quantum regimes^9^. Here, we approach this subject from a more concrete, experimental perspective. For this purpose, we analyze theoretically and experimentally the PINEM discrete sideband spectrum (some recent work can be found, e.g., in^2,3,10^, but they only addressed the case of coherent light) as well as the DLA acceleration spectrum and show that their spectrum implies the wave-particle duality of free electrons when interacting with light. We wish to investigate the various regimes when electrons and photons are coupled to classify in which cases they can be regarded as ‘classical’ or ‘quantum’ measuring pointers. In particular, we study the transition process between the two regimes. In light of the current experimental capabilities of manipulating electrons and photons, the quantitative wave-particle duality of electrons and the quantum-to-classical transitions of photons are both controllable in ultrafast transmission electron microscopy (UTEM)^1,4,11^ and in quantum light preparation^12^, respectively. In the wavepacket representation with electron wavepacket size (${\Delta }_{z}$), the point-particle-like (‘classical’) picture of free electrons can be defined in the limit ${\Delta }_{z}\to 0$ and conversely, the plane-wave-like (‘quantum’) picture in the opposite limit ${\Delta }_{z}\to \infty$. Similarly, the photon state holds its own quantum-to-classical transition. For concreteness, the single-photon-added coherent state enables us to continuously tune the photon system from a coherent state (representing quantum states in their ‘classical’ limit) to a single Fock number state (which we take as a uniquely ‘quantum’ state)^12,13^. Note that, indeed, the coherent state delineates the border regarding the classicality and quantumness of photon states from different perspectives^14^. We thus define a parameterized photon state as the basis for possible investigation of the fuzzy border that may separate the ‘quantum’ from ‘classical’ regimes in the above sense, utilizing the coupling with a single electron wavepacket as a measuring pointer. (We note that the above characterization of classical and quantum states is explicitly tailored to the analysis of interactions between free electrons and photons. Indeed, other notions of classicality could be found in literature).

To be specific, we represent the ‘quantum-to-classical’ transition of photon states using the photon-added coherent state and the ‘particle-to-wave’ electron state using a Gaussian wavepacket. The initially prepared photon and electron states are respectively given by

1 $$\begin{array}{c}|\alpha ,\nu {{\rangle }}=\frac{{\left({a}^{\dagger }\right)}^{\nu }|\alpha {{\rangle }}}{\sqrt{\nu !{L}_{\nu }\left(-{\left|\alpha \right|}^{2}\right)}}\\ |{{\psi }}{{\rangle }}=\int {dp}\,{c}_{p}^{(0)}{|p}{{\rangle }}\end{array}$$

**Fig. 1:**
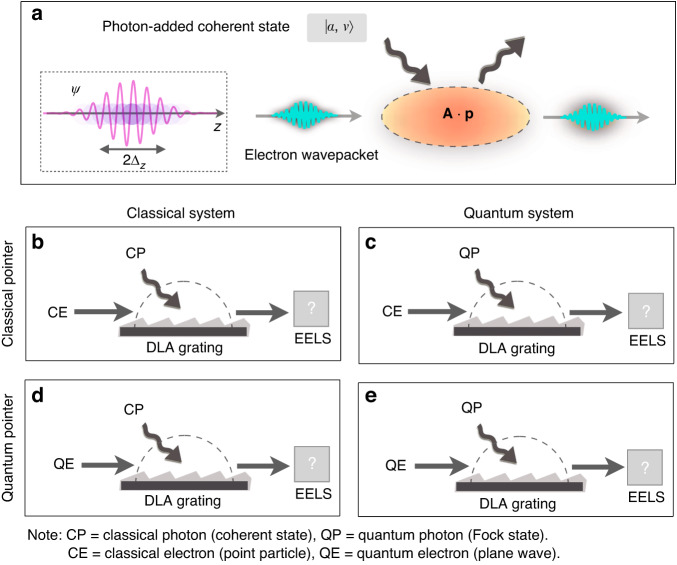
The quantum and classical measurement schemes of free electron–photon interactions. **a** We treat these light–matter interactions using the tools and terminology of measurement theory. In this respect, the measuring pointer is the outgoing electron, and the measured system is the pre-prepared photon state (without post-selection), with a coupling strength *g* between the system and the pointer. **b**–**e** Four combinations of free electron and photon interactions are schematically presented in the classical and quantum measurement regimes, corresponding to the various initial states of the electrons and photons. The readout of the measuring pointer is the electron energy loss spectrum (EELS). The classical photon (CP) in a coherent state and quantum photon (QP) in a Fock state are defined as two opposite limits of the photon-added coherent state $|\alpha ,\nu {\rangle }$, where ${\rm{\nu }}=0$ and ${\rm{\alpha }}=0$, respectively. The classical electron (CE) and quantum electron (QE) are defined as the wavepacket representations in the point-particle and plane-wave limits, respectively (all possibilities are quantitatively described in Eq. (1))

The photon-added coherent state reduces to the limit of Fock or coherent state for the parameters $\alpha \to 0$ or $\nu \to 0$, respectively, with ${L}_{\nu }$ being the Laguerre polynomial of (integer) order $\nu$, ${a}^{\dagger }$ is the photon creation operation, and all other photon indices are suppressed for simplicity. Such a photon state was theoretically proposed by Agarwal and Tara^13^ and later experimentally realized by Zavatta et al. ^12^. The normalized Gaussian component of a free electron wavefunction is

$${c}_{p}^{\left(0\right)}={\left(2{\rm{\pi }}{\Delta }_{p}^{2}\right)}^{-1/4}\exp (-{\left(p-{p}_{0}\right)}^{2}/4{\Delta }_{p}^{2})$$

with ${\Delta }_{{\rm{p}}}$ the unchirped momentum uncertainty, and ${p}_{0}$ the average momentum, respectively. Note that the electron wavepacket is only defined in a longitudinal dimension (1D), where the electron’s initial momentum distribution is readily obtained as ${\rho }^{\left(0\right)}\left(p\right)={\left|{c}_{p}^{\left(0\right)}\right|}^{2}$. Following the standard procedure of measurement proposed by von Neumann^15^, we can study the quantum-to-classical transitions of free electron–photon interactions. As always, the coupling introduced by the von Neumann measurement Hamiltonian creates a variable-strength entanglement between the systems, which allows one to make inferences about one when subjecting the other to a projective measurement. As a testing bed of this measurement-based approach, we shall classify the possible interactions as shown in Fig. 1b–e: (I) A classical point-particle electron coupling with ‘classical’ photon coherent state; (II) a classical point-particle electron coupling with ‘quantum’ photon Fock state; (III) a quantum plane wave electron coupling with classical photon; and (IV) a quantum plane-wave electron coupling with quantum photon.

Next, we assume that the coupling between the classical electron (${\Delta }_{{\rm{z}}}=\frac{{\hslash } }{2{\Delta }_{{\rm{p}}}}\to 0$) and the classical photon ($\nu \to 0$ but $\alpha\, \ne\, 0$) can be simplified into the canonical Hamilton equations $\dot{z}=p/{\gamma }_{0}m,\dot{p}=e{E}_{c}\cos \left(\omega t-{q}_{z}z\left(t\right)+{\phi }_{0}\right)$, which in Newtonian mechanics describe a charged point-particle ($-e$) moving in the presence of a monochromatic traveling electromagnetic field (laser, or microwave field) with an electric component $E={E}_{c}\cos \left(\omega t-{q}_{z}z\left(t\right)+{\phi }_{0}\right)$ having the optical frequency ${\rm{\omega }}$ and the z component of the wave vector ${{\rm{q}}}_{{\rm{z}}}$ along the propagation direction. With the short-time approximation $z(t)={v}_{0}t$, we expect that the point-particle momentum transfer can be thus reduced to $\Delta {\text{p}}_{\text{point}}=-e{E}_{c}L/{v}_{0}\,\text{sinc}\left(\frac{\bar{{{\theta }}}}{2}\right)\cos \left(\frac{\bar{{{\theta }}}}{2}+{{{\phi }}}_{0}\right)$, in which the synchronization condition (also called phase matching condition) is $\bar{{{\theta }}}=\left(\omega /{v}_{0}-{q}_{z}\right)L$, $L$ is the interaction length and ${v}_{0}$ is the initial velocity of the electron. This is the well-known linear acceleration formula in classical accelerator physics, as well as in the inverse Smith–Purcell effect^16^, or the dielectric laser accelerator (DLA)^6,7^ and free electron lasers^10^. In addition, it indicates that the emergence of ‘classicality’ in our measurement setup requires both the classical conditions of ‘point-particle-like’ electron and photon at a coherent state, as shown in Fig. 1b.

**Fig. 2:**
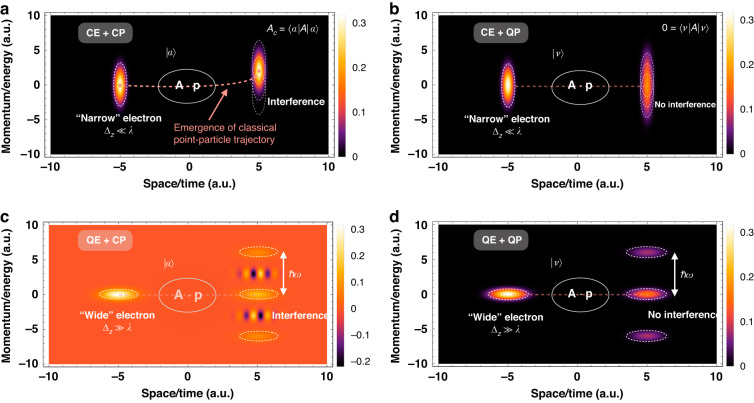
Illustration of four measurement regimes of free electron–photon interactions in phase-space representation. The electrons are presented using Wigner functions corresponding to the specified measurement cases as shown in Fig. 1. **a** Classical electron (CE) interacts with a classical photon (CP) (point-particle ${\Delta }_{z}\ll \lambda ,$ and coherent state $|\alpha {\rangle }$); **b** CE interacts with a quantum photon (QP) (${\Delta }_{z}\ll \lambda ,$ and Fock state $|\nu \rangle$); **c** quantum electron (QE) interacts with a CP (plane-wave ${\Delta }_{z}\gg \lambda ,|\alpha {\rangle}$); **d** QE interacts with a QP (${\Delta }_{z}\gg \lambda ,|\nu {\rangle}$). Crucially, among them, only the case **a** “CE + CP” gives rise to the weak measurement and classical point-particle trajectory in electrodynamics. One can obtain the net transfer as a weak value and observe the emergent classical electron dynamics obeying a classical trajectory with a certain position and momentum in phase space, as shown in (**a**)

This classical acceleration formula offers a hint of how to quantum-mechanically measure the electromagnetic field operators (e.g., the vector potential **A**) via a moving electron wavepacket as a measuring pointer coupled to the measured photonic system. It will be shown how to calculate the classical particle acceleration within the von Neumann measurement scheme^15^ as a result of the electron–photon coupling. From the perspective of weak measurement^17^, we will see below that the momentum transfer of the pointer after interaction corresponds to the weak value of the vector potential (**A**) of the photonic system. This applies to the configuration of a classical electron pointer coupled to a classical photon system (Fig. 1b). In the other three configurations, the electron–photon couplings indicate a quantum (strong) projective measurement. As a result, the system-pointer measurement inevitably falls into the ‘strong’ category involving a significant momentum change with a subsequent ‘wavefunction collapse’, regardless of whether the electron or photon state falls in the quantum regime (Fig. 1c–e). The classification of four measurement regimes will indicate in the following sections how only a quantum weak measurement can lead to classical particle acceleration (Figs. 1b and 2a), thereby possibly implying, in general, how classical electrodynamics may emerge from a full quantum treatment.

Additionally, in the transition from weak to projective mee coupling between the classic measurements, we notice that the identities of electron and photon are reciprocal in the following sense: which is the system and which is the pointer depends on the detection and post-selection configuration of electrons and photons. This underlying reciprocity leads to the system-pointer duality that will be discussed towards the end of this work.

The main novelty of the work relies on the presentation of a unified scheme for analyzing interactions between photons and free electrons, addressing both their classical and quantum regimes, as well as the interesting quantum-to-classical transition. The proposed scheme employs tools from quantum measurement theory and therefore makes it easy to draw the line between weak and strong (projective) measurements in light–matter interactions. In particular, two limits of our theoretical prediction are experimentally tested here with recent UTEM^11^ and DLA^18^ setups.

The rest of the manuscript is organized as follows. In the next two sections, we differentiate between the four basic types of electron–photon couplings, addressing inter alia the emergence of point-particle trajectories. We then analyze the expectation-valued electron spectrum and weak-valued spectrum in our measurement transition theory and find that the classical electron momentum transfer can be viewed as an outcome of a weak measurement in the next two sections. We then verify our theoretical predictions using experimental results obtained in UTEM and DLA setups. Finally, we address the advances of post-selection in light–electron interactions and further clarify the significance of classical-to-quantum transitions in our measurement-based theory.

### Classical photons in a coherent state

Our analysis of measurement is based on the perturbative solution of the relativistically modified Schrödinger equation^10,19–21^ for a free electron wavefunction and a quantized radiation field. Following the standard QED treatment (see the SI file), we expand the initial wavefunction in terms of the quantum continuous numbers *p* of the electron state and the Fock number-occupation state of the photon, which is given by ${|i}{{\rangle }}=\sum _{p,\nu }{c}_{p,\nu }^{\left(0\right)}{e}^{-i{E}_{p}t/{\hslash} }{|p},\nu {{\rangle }}$, where ${c}_{p,\nu }^{\left(0\right)}$ is the component of the combined basis ${|p},\nu {\rangle }={|p}{\rangle }\bigotimes |\nu {\rangle }$.

The net energy transfer as the pointer shift in the electron spectrum is obtained in first-order perturbation theory as $\Delta E=\sum _{p,\nu }{\left|{c}_{p,\nu }^{\left(0\right)}+{c}_{p,\nu }^{\left(1\right)(e)}+{c}_{p,\nu }^{\left(1\right)(a)}\right|}^{2}\left({E}_{p}-{E}_{0}\right)$ where the initial electron energy ${E}_{0}=\sum _{p,\nu }{\left|{c}_{p,\nu }^{\left(0\right)}\right|}^{2}{E}_{p}$^10–12^. In our quantum treatment of the initial electron–photon state as given by ${c}_{p,\nu }^{\left(0\right)}={c}_{p}^{\left(0\right)}{c}_{\nu }^{\left(0\right)}$, we consider the initial electron wavepacket of the unchirped Gaussian distribution (Eq. 1) combined with a coherent photon state, where ${\nu }_{0}=\sum _{\nu }{\nu \left|{c}_{\nu }^{\left(0\right)}\right|}^{2}$ is the total photon number. Substituting into the formula ($\Delta E$), one can obtain the explicit energy transfer with two parts ($\Delta {E}=\Delta {{E}}^{(1)}+\Delta {{E}}^{(2)}$)^9^:

2 $$\begin{array}{c}\Delta {E}^{\left(1\right)}=\Delta {{E}}_{{\rm{point}}}{e}^{-\frac{{\Gamma }^{2}}{2}}\\ \Delta {E}^{\left(2\right)}=-{\widetilde{{{{\Upsilon }}}}}^{2}{{\hslash }}\omega \,{\rm{sinc}}^{2}\left(\frac{\bar{{\rm{\theta }}}}{2}\right)\end{array}$$

where $\Delta {{{E}}}_{{\rm{point}}}={v}_{0}\Delta {{{p}}}_{{\rm{point}}}=-{\rm{e}}{{{E}}}_{{\rm{c}}}{{L\; {\rm{sinc}}}}\left(\frac{\bar{{{\theta }}}}{2}\right)\cos \left(\frac{\bar{{{\theta }}}}{2}+{{{\phi }}}_{0}\right)$ and the normalized photon exchange coefficient of spontaneous emission is defined as $\widetilde{{\rm{{\Upsilon }}}}=e{\widetilde{E}}_{q}L/4{\hslash } \omega$, corresponding to the PINEM near-field factor as $g=2\sqrt{{\nu }_{0}}\,\widetilde{{\rm{{\Upsilon }}}}$ (see the SI file). Note that the relation $\sqrt{{\nu }_{0}}={\langle }\sqrt{{\nu }_{0}}|{a}_{\nu }|\sqrt{{\nu }_{0}}{\rangle }$ is taken for the coherent state ($|\sqrt{{\nu }_{0}}{\rangle }$). A significant pointer-specific extinction parameter ${e}^{-{\Gamma }^{2}/2}$ is found in the phase-dependent energy transfer (2), with a decay parameter given by

3 $$\Gamma =\frac{2\pi }{\beta }\left(\frac{{\Delta }_{{\rm{z}}}}{\lambda }\right)=\frac{{{\hslash }}\omega }{{v}_{0}{\Delta }_{{\rm{p}}}}$$

with $\beta ={v}_{0}/c$. The extinction parameter demonstrates that it is the pre-interaction history-dependent wavepacket size of a free-electron wavepacket that has a physical effect in its interaction with coherent light and acts as the measuring pointer.

Now, we are able to discuss quantitatively the classical point-particle and quantum plane-wave limits of electron wavepacket energy transfer in the interaction with the quantized photon state of light, as shown in Fig. 1b, d. The particle-to-wave transition of the electron–photon interaction in measuring electron energy loss spectroscopy (EELS) is shown in Fig. 2a. The appearance of ‘classicality’ corresponds to the case where the photon distribution becomes a coherent state describing the ‘classical’ electromagnetic field, and the condition ${e}^{-{\Gamma }^{2}/2}\to 1$ is satisfied, which means that the electron wavefunction looks like a point-like particle with wavepacket size smaller than the wavelength: ${\Delta }_{{\rm{z}}}\ll \lambda$ (“narrow” electron). Indeed, the wavepacket-dependent transfer when the wavepacket size is comparable to the wavelength is given by $\Delta E=\Delta {{E}}_{\text{point}}{e}^{-{\Gamma }^{2}/2}$. Therefore, it explains the emergence of classical point-particle trajectory in the free electron–photon setup of “CE + CP”. The decay parameter ($\Gamma$) implies the measurability of the electron wavepacket size near the classical particle-like regime.

On the other hand, the plane-wave limit of electron can be directly defined as ${e}^{-{\Gamma }^{2}/2}\to 0$ in which case the energy transfer has only the contribution of the phase-independent term ($\Delta {E}^{(2)}$), as shown in Fig. 2c. Note that even in the classical limit, the phase-independent term still has a universal non-vanishing noise contribution in the form of vacuum fluctuations. This phase-independent term ($\Delta {E}^{(2)}$) relates to the vacuum expectation value, which acts as quantum noise of spontaneous fluctuation in our electron–photon coupling measurements^10,20^. Therefore, the phase-dependent term ($\Delta {E}^{(1)}$) reduces to the classical particle acceleration but is measurable only if the spontaneous vacuum fluctuation is negligible: $\Delta {E}^{(1)}\gg \Delta {E}^{(2)}$ under ${\nu }_{0}\gg 1$.

### Quantum photon in a Fock state

In contrast, the single Fock state of light corresponds to the photon-added coherent state (Eq. 1), obeying the condition $\alpha \to 0$, i.e., ${c}_{\nu }^{\left(0\right)}={\delta }_{\nu ,{\nu }_{0}}$. When inspecting the wavepacket energy transfer expression, it appears that, similarly to the case of spontaneous emission, there is no Fock state stimulated energy transfer due to the orthogonality relations ${\langle }{\nu }_{0}|{a}_{\nu }|{\nu }_{0}{\rangle }={\langle }{\nu }_{0}|{a}_{\nu }^{\dagger }|{\nu }_{0}{\rangle }=0$. Therefore, one obtains the total energy transfer, $\Delta {{E}}=\Delta {{{E}}}^{(1)}+\Delta {{{E}}}^{(2)}=-{\widetilde{{\rm{{\Upsilon }}}}}^{2}{\hslash }\, \omega\, {\rm{sinc}}^{2}\left(\frac{\bar{{\rm{\theta }}}}{2}\right)$. There is no stimulated radiative interaction as a result of the coupling to the quantum light (radiation wave) in Fig. 1c, e. However, this is not very surprising since the initial single Fock state ($|{\nu }_{0}{\rangle }$) is orthogonal to the emitted and absorbed photon state ($|{\nu }_{0}\pm 1{\rangle }$), so the first-order phase-dependent interference term has no contribution. Therefore, the phase-space descriptions of an electron interacting with a quantum light source, depicted in Fig. 2b, d present either momentum broadening (in the classical electron case) or distinct quantum sidebands (in the quantum electron case), without the contribution of quantum interference between photon sidebands depicted in Fig. 2c in the case of classical light quantum electron case^22^. This is a result of quantum entanglement between two different Fock states. Note that the second term ($\Delta {{{E}}}^{(2)}$) still produces the wavepacket-independent spontaneous vacuum fluctuations as an inevitable source of quantum noise in the observation of EELS in the quantum light case^20^, same as in the case of classical light (Eq. 2).

### Weak measurement versus projective measurement

Let us focus now on the EELS observation of the final electron wavefunction after the interaction. When a quantum electron pointer is coupled to the photon system, the photon-induced outgoing electron momentum distribution is then given by integrating out all photonic degrees of freedom that is, ${\rho }^{\left(f\right)}(p)=\sum _{\nu }{\left|{c}_{p,\nu }^{\left(0\right)}+{c}_{p,\nu }^{\left(1\right)(e)}+{c}_{p,\nu }^{\left(1\right)(a)}\right|}^{2}$. Let us find the EELS measurement pictures in the two aforementioned limits.

**Fig. 3:**
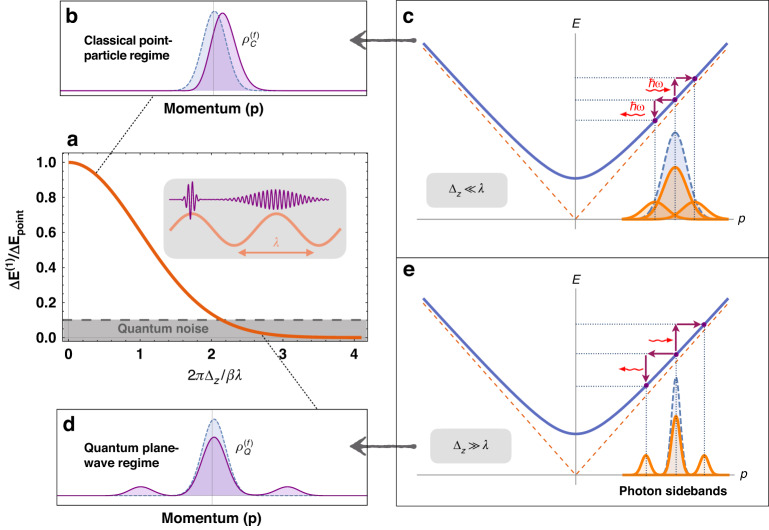
Quantum-to-classical measurement transition of the electron wavepacket pointer when coupled to a photon coherent state. **b**, **c** Under the coupling condition with a narrow electron ${\Delta }_{{\rm{z}}}\ll \lambda$, the photon sidebands overlap, and its asymmetric interference would result in a redistribution of the final electron spectrum with a net momentum transfer. **d**, **c** In contrast, under the condition with wide electron ${\Delta }_{{\rm{z}}}\gg \lambda$, these sidebands are distinctly separated, resulting in a spectrum that is discrete. **a** The two limits of particle-like (**b**, **c**) and wave-like (**d**, **e**) pictures of the electron pointer in light–matter interactions correspond to the classical (weak) measurement (particle accelerator) and quantum (projective) measurement (PINEM), respectively. The exact expressions of the final momentum distributions are presented in the text

First, in the point-particle limit ${\Delta }_{{\rm{z}}}\ll \lambda$ ^19,23^, necessarily, the initial momentum distribution exceeds the quantum momentum recoil ${\Delta }_{{\rm{p}}}\, > \,{\hslash }\omega /{v}_{0}$ and hence the final momentum distribution after interaction with the classical photon is: ${\rho }_{C}^{\left(f\right)}\left(p\right)={\rho }^{\left(0\right)}(p-\Delta {p}^{(1)})$, where the momentum shift is $\Delta {p}^{(1)}=\Delta {\text{p}}_{\text{point}}{e}^{-{\Gamma }^{2}/2}$ (also corresponding to the energy transfer $\Delta {{{E}}}^{(1)}$ in Eq. 2). As shown in Fig. 3b, c, the emission and absorption terms overlap with the initial wavepacket momentum distribution and contribute the asymmetrical interference effects with opposite sign, which leads to the momentum shift in the classical point-particle regime. The final momentum distribution of the electron pointer is then reshaped, displaying a net momentum shift of small acceleration, as shown in Fig. 3b, c, where we ignore the spontaneous term in the weak-field coupling $e{E}_{c}L/{\hslash }\omega < 1$. Except for the universal transition factor ${e}^{-{\Gamma }^{2}/2}$, the acceleration/deceleration of the electron wavepacket depends on the synchronism detuning parameter $\bar{{\rm{\theta }}}$ and the relative phase ${{\rm{\phi }}}_{0}$, similar to a charged point-particle moving in the presence of a classical electromagnetic field ($\Delta {\text{p}}_{\text{point}}$) in the classical limit of interaction between ‘particle-like’ electron and ‘classical’ photon (Figs. 1b and 2a). This interaction picture of electron–photon coupling leads to the classical measurement or classical electrodynamics and also to the weak measurement, as displayed in Fig. 3b.

Next, in the plane-wave (quantum electron) limit ${\Delta }_{{\rm{z}}}\gg \lambda$, corresponding to the large recoil condition ${\Delta }_{{\rm{p}}}\, <\, {\hslash }\omega /{v}_{0}$ (i.e., the criterion of projective measurement), the interference terms between sidebands diminish, and the scattered components dominate, resulting in a final PINEM-kind spectrum of the momentum distribution, as shown in Fig. 3d, e: ${\rho }_{Q}^{\left(f\right)}\left(p\right)=\left(1-2{{\rm{{\Upsilon }}}}^{2}{\rm{sinc}}^{2}\left(\frac{\bar{{\rm{\theta }}}}{2}\right)\right){\rho }^{\left(0\right)}\left(p\right)+{{\rm{{\Upsilon }}}}^{2}{\rm{sinc}}^{2}\left(\frac{\bar{{\rm{\theta }}}}{2}\right)\left({\rho }^{\left(0\right)}\left(p-{\hslash }\omega /{v}_{0}\right)+{\rho }^{\left(0\right)}(p+{\hslash }\omega /{v}_{0})\right)$ where ${\rm{{\Upsilon }}}=e{E}_{c}L/4{\hslash }\omega$ and we ignore the spontaneous contribution to the emission term with the approximation ${\nu }_{0}\gg 1$. The last two scattering terms represent symmetric photon-sideband spaced by ${\hslash }\omega /{v}_{0}$ on both sides of the central momentum ${p}_{0}$ of the wavepacket as displayed in Fig. 3e. This quantum measurement result is the same as the multiple sidebands electron energy gain/loss spectrum in PINEM experiments, in which the higher-order sidebands relate to multiple-photon emission and absorption processes^1,2^.

For the Fock state of the photon system, the phase-dependent interference terms disappear due to the orthogonality and thus lead to the same final projective momentum distribution as the measurement in the plane-wave limit of electron (with coherent light), regardless of the electron’s wavefunction profile in the classical or quantum limit. For the other three electron–photon couplings in Fig. 1c–e, either quantum electron or quantum photon corresponds to the final projective momentum distribution with no net momentum transfer $\Delta p\,=\,\int {\rho }^{\left(f\right)}\left(p\right)p\,{dp}\,=\,0$ (i.e., $\Delta {{E}}\,=\,0$), which implies no classical measurement for these three system-pointer interaction configurations.

### Is the net energy transfer a weak value?

As demonstrated in Fig. 3b, d, we find that the projective measurement^15^ corresponds to the electron spectrum with discrete photon-sidebands of PINEM (Fig. 3d), and the weak measurement^17^ to the acceleration spectrum with central momentum shift (Fig. 3b). Moreover, the energy/momentum transfer is proportional to the classical electric field given by ${\boldsymbol{E}}=-\left\langle \partial {\boldsymbol{A}}/\partial t\right\rangle$ in the Coulomb gauge $\nabla \cdot {\boldsymbol{A}}=0$. Our results seem to depend on the gauge choice of the vector potential ${\boldsymbol{A}}$, but in our case, it is just ${\boldsymbol{A}}{\boldsymbol{=}}\int {\boldsymbol{E}}\,{dt}$, i.e., completely defined by the physically gauge-independent electric field (up to a meaningless integration constant). Such gauge independence was shown to arise when performing a weak measurement of the vector potential and measuring the Berry phase^24,25^. Thus, the classical point-particle acceleration is an effective weak value of the vector potential in the formalism of weak measurement,

4 $$\Delta {{\rm{p}}}_{{\rm{point}}}\propto {A}_{w}\equiv \frac{{\langle }\beta ,{\nu }^{{\prime} }{|}{\boldsymbol{A}{|}}\alpha ,\nu {{\rangle }}}{{\langle }\beta ,{\nu }^{{\prime} }|\alpha ,\nu {\rangle }}$$

where the pre- and post-selected photon states are both defined as photon-added coherent states (1). Note that this definition of the vector-potential weak value is applicable only if there is no time evolution of the photon system (except for the measurement process) or, effectively, in short-time approximation. Two typical examples are considered with fixing the pre-selection and post-selection at the ‘classical’ or ‘quantum’ photon state, respectively: $|\alpha ,\nu \rangle=|\beta ,\nu ^{\prime}\rangle =|\sqrt{{\nu }_{0}},0\rangle =|\sqrt{{\nu }_{0}}\rangle$ (Fig. 1b, c); $|\alpha ,\nu {\rangle }=|\beta ,\nu {\prime} {\rangle }=|0,{\nu }_{0}{\rangle }=|{\nu }_{0}{\rangle }$ (Fig. 1d, e). Also, these examples correspond to the electron energy transfers (i.e., Eq. 2), as we discussed in the previous two sections.

Now we describe the electron coupling process with a classical-like photon system in the scheme of weak measurement^17,26^, which is given by

$$\begin{array}{c}\underbrace{\langle \beta ,\nu ^{\prime} |}_{{\rm{post}}-{\rm{selection}}}\,\underbrace{{\mathscr{T}}\exp \left(-\frac{i}{\hslash }\mathop{\int }\limits_{0}^{L/{v}_{0}}{H}_{I}(t)dt\right)}_{{\rm{electron}}-{\rm{photon}}\,{\rm{coupling}}}\underbrace{|\alpha ,\nu \rangle \otimes |\psi \rangle }_{{\rm{pre}}-{\rm{selection}}}\\ =\langle \beta ,\nu ^{\prime} |\left(1+\frac{ie}{{\gamma }_{0}m\hslash }\mathop{\int}\limits_{0}^{L/{v}_{0}}{\boldsymbol{A}}(t)\cdot {\boldsymbol{p}}\,dt\right)|\alpha ,\nu \rangle \otimes |\psi \rangle \\ \begin{array}{c}\left.=\langle \beta ,\nu ^{\prime} |\alpha ,\nu \rangle \otimes \left|\psi \left(z+\frac{e}{{\gamma }_{0}m}\mathop{\int }\limits_{0}^{L/{v}_{0}}{A}_{w}(t)dt\right)\right\rangle \right.\\ ={e}^{-{|\beta -\alpha |}^{2}/2}|\psi (z-\Delta z)\rangle \end{array}\end{array}$$

where we employed the relation $\langle\beta ,0{|}\alpha ,0\rangle ={e}^{-{\left|\beta -\alpha \right|}^{2}/2}$ for coherent states with real numbers $\alpha ,\beta$. The measuring electron pointer is assumed to be a Gaussian wavepacket in coordinate space (*z*) corresponding to its momentum component (Eq. 1, i.e., $|\psi \rangle$). The final spatial shift of the electron pointer is thus $\Delta z=-\frac{e}{{\gamma }_{0}m}{\mathop{\int }\nolimits_{0}^{L/{v}_{0}}}{A}_{w}\left(t\right){dt}$ and the corresponding momentum transfer is approximated instantaneously as $\Delta p={\gamma }_{0}m\left(\frac{\Delta z}{\Delta t}\right)=-e{\bar{A}}_{w}$, which confirms the equivalence between the quantum wavepacket momentum transfer in the point-particle limit and the time-averaged weak value of vector potential in the short-time approximation.

At this point, we would like to mention that our measurement-based theory of electron–photon interactions are one-dimensional. However, our assumptions regarding the electron and light fields can be extended toward a three-dimensional QED model in which the transverse profiles of both the electron wavepacket and light field are incorporated. In such as description, the spin and orbital angular momentums of both electrons and photons would have a profound role and could lead to new effects, e.g., ^27,28^. (Note that the weak value ${A}_{w}$ is, in general, a complex number. Conceptually, weak values appear to suggest an approach to describing quantum systems with two boundary conditions (pre- and post-selection), see e.g., ^26,29^. However, ${A}_{w}$ is real in our case because the photon state is a coherent state, an eigenstate of the vector potential, thus, we expect that ${A}_{w}=2\mathrm{Re}\{{A}^{(-)}\}$, where ${A}^{(\mp )}$ are negative and positive frequency components of the vector potential, respectively).

### Experimental verification

**Fig. 4:**
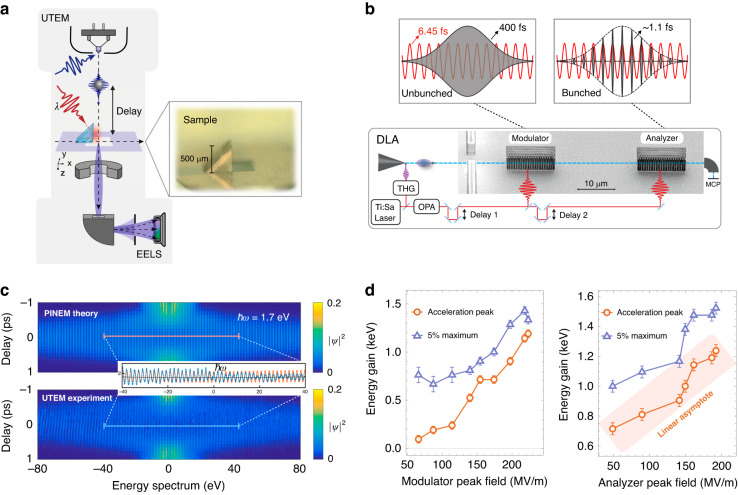
Experimental demonstrations of free-electron–photon measurements in the quantum (PINEM) and the classical (DLA) cases. **a** The schematic setup of UTEM with a prism-mediated laser-near-field electron interaction used to obtain the PINEM spectrum. **b** The schematic setup of the DLA-based experiment with two gratings (modulator and analyzer) was used to obtain net electron acceleration. **c** The symmetric energy spectrum with sideband spacing of ${\hslash }\omega$(1.7eV) indicates the quantum regime of the electron–photon interaction. The data (bottom panel) matches well with the PINEM theory of wide electrons in the plane-wave limit, the simulation results of which are shown in the top panel. **d** The left panel shows the net energy transfer as a function of the modulator peak field and for a constant analyzer peak field of 207 MV/m. Figure S2 of the Supplementary Information shows simulation data with a similar behavior, hence this behavior shows the transition from unbunched to bunched electron beams. With a maximum modulator field strength of 224 MV/m here, the bunched pulse duration is around 1.1 fs, considerably smaller than the optical cycle (6.45 fs), thus showing the point-particle classicality of the bunched electrons. The right panel shows the energy gain for both the acceleration peak and the maximum 5% (see Fig. S4 in the SI file) for a modulator peak field of 224 MV/m. Both depend linearly on the analyzer’s field strength, demonstrating the weak value measurement of the photon system. The “5% maximum” is the maximum peak acceleration at 5% of the count rate

To verify the two measurement limits from quantum to classical, let us compare our theory with specific experimental results of PINEM in a UTEM and electron spectra in DLA. The two setups are depicted in Fig. 4a, b (see also^11,18,30,31^). Let us clarify that from the point of view of our theoretical one-dimensional model, there is no difference between the prism-based setup (see the inset of Fig. 4c) and the periodic rod structures of Fig. 4b. The relevant classical light field $E(z,t)$ is in the first case the evanescent near field of the laser-illuminated dielectric prism, and, in the second case, a slow-wave space harmonic of a Floquet mode in the periodic rods of the DLA structure. The derived extended electron–photon interaction is the same in both setups as long as the electron and the wave are synchronized or slightly desynchronized with the same detuning parameter $\bar{{\rm{\theta }}}$ ^10^. Here we mention recent work^22,32^ that, from a different view of photons, studied the quantum-to-classical transition of the photon statistics of quantum light sources on free-electron–light interactions in a UTEM setup with the DLA device. Notice that femtosecond light pulses are employed instead of a continuous-wave laser, and indeed, the letter may benefit from three features. First, the application of the UV pulse generates an ultrashort electron pulse. Second, the duration of the IR pulse controls well the effective interaction time of the electron–photon coupling. Third, the femtosecond pulses allow us to fine-tune the delay between the electron pulse and the IR pulse.

In the first case (Fig. 4a), the measured spectrum of the interacting wide electron wavepacket depicts a pattern of multiple discrete sidebands with ${\hslash }\omega$ spacing. This is explained in terms of PINEM theory in the multiphoton coupling regime (Section E in the SI file). Indeed, the width of the measured spectrum with the discrete photon sidebands can be extended to the range of 1 keV and shows no net linear acceleration, even in the case of extremely strong field and non-perturbative laser–electron interaction.

We compare the interaction with the prism in Fig. 4a to the interaction with the second periodic rod structure (the analyzer) in Fig. 4b. The crucial difference is that in the latter case, the electrons arrive at the interaction region in a density-modulated state: The optical near-fields in the first periodic rod structure (the modulator) led to an energy modulation without net energy transfer. This, together with the free drift of the electron wavepackets, generates a train of attosecond-bunched electrons when they arrive at the analyzer (see also^18^). The periodically bunched electrons display a minimal bunch duration of ~1.1 fs, which is smaller than the synchronized optical cycle (6.45 fs). Hence, this situation corresponds to an optical system interacting with a train of electron-measuring pointers. In this case, the analyzer can perform a weak measurement of the optical field system when the modulator pre-bunches the electrons, and thus, classical acceleration is expected for the bunched electrons, as shown in Fig. 4d. We can interpret this as a weak measurement of the optical field system as proposed above. The right panel of Fig. 4d shows the linear dependence of the energy gain as a function of the analyzer’s field strength for the optimally bunched electrons. We can interpret this as the modulator creating a transition from projective to weak measurement. Furthermore, like in the DLA setup, we can suggest an extended PINEM experiment to have two-stage near-field interactions phase-matched such that the first stage produces attosecond bunching at the second stage, where then a large transfer may also be observed. We explain how the pre-bunched electron pulses in the PINEM regime can constitute a weak measurement in the Supplementary Information (Figs. S1–S3 in the SI file). Additionally, spectrograms of the modulated electron density as a function of delay time are given in Fig. S4.

### Post-selection on electrons or photons

**Fig. 5:**
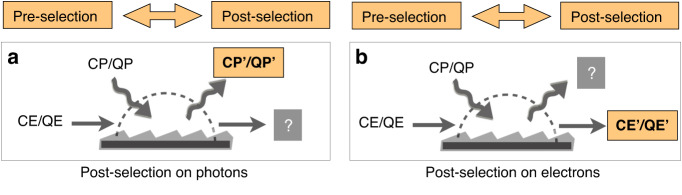
The weak-valued electron–photon interaction with pre-/post-selection on photons and electrons. The pre- and post-selections are performed on (**a**) the photons or (**b**) the electrons as the measured system, and the rest acts as the measuring pointer in quantum-to-classical measurement schemes

Let us discuss the post-selection of the electron–photon states after interaction in terms of measurement theory. Two types of ‘weak-valued’ electron–photon couplings are schematically shown in Fig. 5. In the reciprocal system-pointer setup of light–matter interaction, the electron can be the measured system, and the photon is then the measuring pointer. If we are able to pre- and post-select the electron wavefunction, detection of the photon radiation rate ($\Delta \nu$) then leads to a shift of the photon pointer, being the measuring pointer, as compared to the measurement of the momentum operator of the electron. In a recent work^10,20^, the reciprocal relation between photon radiation and electron acceleration is demonstrated to be $\Delta \nu +\Delta E/{\hslash }\omega =0$, which brings a correspondence between the electron spectrum and photon spectrum that conserves the photon exchange in all measurement schemes. This ‘acceleration/radiation correspondence’ (ARC) relation^20^ connects the final measurements of the photon and electron spectrum with/without post-selection as a demonstration of the ‘system-pointer’ dualism. This setup of weak measurements resembles the pre- and post-selection of atomic states coupled with photons, as proposed recently by Aharonov et al. ^33^.

Note that our quantum-to-classical measurement theory is entirely different from the environment-induced decoherence program^34–36^. Decoherence theory, in which the ‘classicality’ emerges from the natural loss of quantum interference by ‘leakage’ into the environment^34^, does not comprise the contributions of quantum interference and would neither yield wavepacket-dependent energy transfer nor periodic density bunching in the attosecond scale as in^37–39^. Likewise, the environment-induced decoherence cannot produce the classical linear particle acceleration. (Unlike projective measurements, weak measurements maintain the coherence of quantum states^40,41^. Therefore, one would naively expect that the results agreeing with classical intuition would be obtained in a strong projective measurement after averaging overall outcomes. For instance, the average position of a quantum harmonic oscillator in a coherent state reproduces the evolution of a classical harmonic oscillator. In our analysis, we have also demonstrated quantum-to-classical transitions in weak measurements).

### Conclusion

Four kinds of measurement setups of electron–photon interactions were considered in detail, loosely corresponding to ‘classical electron–classical photon’, ‘classical electron–quantum photon’, ‘quantum electron–classical photon’, and ‘quantum electron–quantum photon’. We captured all these interaction types using our unified framework of measurement transition theory, defining all the physics above as a consequence of weak measurement or projective measurement. Then, the transition process was characterized by a universal factor ${e}^{-{\Gamma }^{2}/2}$, which could quantitatively verify our measurement theory in any experiment exhibiting light–matter interactions. Furthermore, our work experimentally reveals the continuous transition from weak to projective measurements, which can also explain the quantum-to-classical transition in common schemes like DLA and PINEM. Future research of these processes may also address structured waves (either light or matter, classical or quantum) as well as additional sources of quantum light and potentially supercontinuum light pulses^42,43^.

In addition, we identified the classical linear point-particle acceleration as the weak value of the vector potential and connected it with the appearance of ‘classicality’ in quantum mechanics. This indicates that weak measurements not only reveal ‘anomalous’ quantum features of quantum physics but also surprisingly describe classical characteristics in the realm of classical electrodynamics. The weak value of the vector potential under suitable pre- and post-selections offers a compelling theoretical framework for investigating the interaction between electron wavefunctions and quantum light sources, such as the superposition of Fock states or squeezed states of light^22,32^. In a recent paper^44^, we have considered, both theoretically and experimentally, the weak-to-strong transition of quantum measurements in trapped ions as a consistent extension of our theoretical framework to generic system-pointer interactions.

## Methods

### UTEM setup

The measurement of PINEM spectra is conducted on a laser-excited right-angle prism sitting in an ultrafast transmission electron microscope (UTEM). The experimental setup is detailed and described in the previous report^11^. Briefly, the UTEM (JEOL-2100P) is operated in nano-beam diffraction (NBD) mode with a 70 µm condenser aperture and 207.2 kV electron acceleration voltage for small and parallel electron probes. The right-angle prism (BK7 glass, *n* = 1.512 at 730 nm wavelength) is placed on a TEM holder inside UTEM, with one of its right-angle faces aligned parallel to the electron beam. To generate the light evanescent field on the prism face, a 730 nm laser beam from a pump femtosecond laser system (Light Conversion) is coupled to the UTEM from a transparent port through an in-column mirror, and finally incident on the prism. Such beam yields phase-matching of electron and evanescent field with a Cherenkov angle of 19.8° In one acquisition, photon-excited electron wave-packets are produced from a cathode illuminated by ultraviolet femtosecond laser pulses from frequency conversion of the pump laser and interact with the evanescent field. Their energy spectra are captured by an electron energy loss spectrometer (EELS) with many minutes of integration time. By changing the delayed time of electron and laser pulses, the two-dimensional map showing the evolution of the interaction as a function of time is revealed in Fig. 4c.

### DLA setup

The ballistic bunching is measured in an ultrafast scanning electron microscope. The pulsed electron beam, triggered from a standard Schottky-emitter by a 167 kHz frequency quadrupled Ytterbium fiber laser, focused to sub-100 nm to enter the channel of the first DLA structure, the modulator. The DLA structures are fabricated from silicon single crystals via deep reactive ion etching (DRIE). The structures tailor the near field when illuminated by the same fiber laser, upconverted to ~2 μm wavelength via an optical parametric amplifier system, to be synchronous with the electrons. The near field interacts in the modulator with the flying electrons and modulates their energy sinusoidally. The subsequent drift between the two structures causes ballistic bunching due to the non-relativistic electrons having different velocities. The spatial distribution is probed in the second structure, the analyzer. The near field of the analyzer interacts with the modulated attosecond-bunched electrons, which leads to a net energy transfer. Finally, the energy transfer is revealed with a magnetic spectrometer. More details can be found in^18,30^.

The theoretical modelling, calculations, Supplementary Figs., and data are available in the SI file.

### Supplementary information

supplementary_materials
