# Supplementary material for: Weak measurements and quantum-to-classical transitions in free electron–photon interactions
Source: Light Sci Appl. 2023 Nov 8;12:267. doi: 10.1038/s41377-023-01292-2 (PMC10632359; doi:10.1038/s41377-023-01292-2)
Supplement: Supplementary file 1 — supplementary_materials [file 41377_2023_1292_MOESM1_ESM.docx]

**Supplementary Information for**

**Weak measurements and quantum-to-classical transitions in free electron-photon interactions**

Yiming Pan^1,6,9†^, Eliahu Cohen^2†^, Ebrahim Karimi^3^, Avraham Gover^4^, Norbert Schönenberger^5^, Tomáš Chlouba^5^, Kangpeng Wang^6^, Saar Nehemia^6^, Peter Hommelhoff^5^, Ido Kaminer^6^ and Yakir Aharonov^7,8^

1. School of Physical Science and Technology and Center for Transformative Science, ShanghaiTech University, Shanghai 200031, China
2. Faculty of Engineering and the Institute of Nanotechnology and Advanced Materials, Bar Ilan University, Ramat Gan 5290002, Israel
3. Department of Physics, University of Ottawa, Ottawa, Ontario, K1N 6N5, Canada
4. Department of Electrical Engineering Physical Electronics, Center for Laser-Matter Interaction (LMI), Tel Aviv University, Ramat Aviv 6997801, Israel
5. Department of Physics, Friedrich-Alexander Universität Erlangen-Nürnberg (FAU), Staudtstraße 1, 91058 Erlangen, Germany
6. Department of Electrical Engineering, Technion, Haifa 3200003, Israel
7. School of Physics and Astronomy, Tel Aviv University, Ramat Aviv 6997801, Israel
8. Institute for Quantum Studies, Chapman University, Orange, CA 92866, USA
9. Department of Physics of Complex Systems, Weizmann Institute of Science, Rehovot 7610001, Israel

**†**Correspondence and requests for materials should be addressed to E.C. (eliahu.cohen@biu.ac.il) and Y.P. (yiming.pan@shanghaitech.edu.cn).

**This PDF file includes:**

Supplementary Text

Figs. S1 to S4

Supplementary Text

1. **The modified ‘relativistic’ Schrödinger equation from the Klein-Gordon equation**

Some of the electron-radiation interaction schemes referred to in the paper (Smith-Purcell radiation, PINEM, FEL, etc.) operate with a relativistic beam; therefore, the use of Schrödinger equation would not be satisfactory for all cases of interest. Since spin effects are not relevant for the present problem, we do not need to use Dirac equation, but rather base our analysis on the Klein-Gordon (KG) equation. Furthermore, following Ref. [11, 9] in the text, we reiterate the derivation of a Schrödinger-like equation out of the KG equation, using the second-order iterative expansion of the free electron energy around its center energy $\varepsilon_{0}=\sqrt{p_{0}^{2}c^{2}+m^{2}c^{4}}$. This expansion reduces the quadratic KG equation into the parabolic Schrödinger equation under the well-satisfied approximation that the initial momentum spread and the momentum change due to the interaction are within the range $\Delta p\ll p_{0}$ .

The Klein-Gordon equation originates from the relativistic energy-momentum dispersion:

$E_{p}^{2}=p^{2}c^{2}+m^{2}c^{4},$ (A1)

where *m* is the electron rest mass, and *c* is the speed of light. To obtain the KG equation, we make the replacements (minimal coupling with electromagnetic radiation) and apply the differential operator on a wavefunction:

(A2)

where *e* is an electron charge. The KG equation can describe the relativistic electrons in most of the considered radiation schemes if spin effects are negligible. If the radiation field is weak, $e\text{A}/mc\ll1$, then excitation of the negative (positron) energy brunch of the dispersion equation is negligible and one can approximate the wavefunction $\psi\left(r,t\right)$with a single quasi-harmonic positive energy wave

(A3)

where $\varepsilon_{0}=\sqrt{p_{0}^{2}c^{2}+m^{2}c^{4}}=\gamma_{0}mc^{2}$, $p_{0}$ the center momentum and $u\left(r,t\right)$ is a slowly varying function of time. Then substitution of Eq. (A3) in (A2) and canceling the fast-varying coefficient , result in

(A4)

This is an exact expression for the slow part function $u\left(r,t\right)$. The first-order approximation of its time derivative is

Iterative substitution of this equation into the exact formula (A4) results in

(A5)

Now the Klein-Gordon equation can be re-expressed in the form of a modified Schrödinger equation,

(A6)

where the effective Hamiltonian is

(A7)

The Hamiltonian can be split into an unperturbed electronic part and a radiative perturbation part $H=H_{0}+H_{I}\left(t\right)$ , where

(A8)

and to first order in the vector potential$\text{A}$,

(A9)

where $v_{0}=p_{0}/\gamma_{0}m=\beta_{0}c$ and $\gamma_{0}=1/\sqrt{1-\beta^{2}}$ is the Lorentz factor, $\beta=\text{v}_{0}/c$ and $m$ is the free electron mass. Note that the coupling term ($\hat{\text{A}}\cdot\text{p}$) has been widely applied to describe numerous kinds of light-matter interactions, like the Smith-Purcell effect, Cherenkov radiation, and transition radiation, regardless of the grating, tip, foil, and nanostructures. For specification in our one-dimensional electron-photon interaction model in a slow-wave structure (such as a grating), we consider a monochromatic laser field with frequency$\omega$, $\hat{\text{A}}=-\frac{1}{2i\omega}\left(\hat{\text{E}}\left(r\right)e^{-i\omega t}-\hat{\text{E}}^{\dagger}\left(r\right)e^{i\omega t}\right),$ where $\hat{\text{A}},\hat{\text{E}}$ are electromagnetic field operators. In our one-dimensional analysis, we assume that the light-electron coupling takes place through an axial slow-wave field component of one of the traveling modes (q): where $\hat{a}_{q}\left(\hat{a}_{q}^{\dagger}\right)$is the annihilation (creation) operator of the photon’s Fock state $|\nu\rangle$ in this quantized mode with wave number *q* and the normalized polarization vector $\text{ε}_{z}$pointing in the direction of propagation *z.*

For the case of our concern,

(A10)

In our present one-dimensional analysis, we assume a longitudinal field component of a slow-wave structure (e.g. a grating) $\tilde{\text{E}}\left(z\right)=E_{c}e^{iq_{z}z}\hat{\text{e}}_{\text{z}}$, neglecting transverse field components, and transverse variation of the field. This modified relativistic Schrödinger equation with the effective Hamiltonians (A8), (A9) is used in the main text for the perturbative solution.

1. **The details of the first-order perturbation analysis**

Following the standard QED treatment (the time-dependent evolution operator $U=\hat{T}\exp\left( -i\int H_{int} dt' \right)$), we expand the initial wavefunction in terms of the quantum continuous numbers *p* of the electron state and the Fock number-occupation state of the photon, which is given by , where $c_{p,\nu}^{(0)}$ is the component of the combined electron and photon state $|p,\nu\rangle$ as given in Eq. (1) and $E_{p}=c\sqrt{m^{2}c^{2}+p^{2}}$. First-order time-dependent perturbation analysis of the Schrödinger equation results in and the interaction Hamiltonian is taken to be $\hat{H}_{I}\left(t\right)\text{=-}\text{e}\hat{\text{A}}\cdot p/\gamma_{0}m$ (see Sec. A). Via second quantization of the vector potential in terms of photon creation and annihilation operators - $\hat{\boldsymbol{A}}\boldsymbol{=A}\left( a_{q}^{\dagger},a_{q} \right)$, we can unitarily split the interaction Hamiltonian into two parts: $H_{int}=H_{int}^{(e)}+H_{int}^{(a)}$ , in which $H_{int}^{(e, a)}$correspond to the reciprocal photon emission (e) and absorption (a) respectively.

By integrating in the time domain to infinity, the emission and absorption processes of the first order perturbed coefficients are given by [9] as follows,, where the matrix elements $H_{I}^{(\mathrm{e},\mathrm{a})}$ correspond to the emission (e) and absorption (a) parts of the interaction Hamiltonian, respectively. Note that the scattered components $c_{p,\nu}^{\left( 1 \right)\left( e,a \right)}$ represent the reciprocal momentum conserving processes through emission or absorption of a single-photon and momentum recoils, relating to the incoming component $c_{p,\nu}^{\left( 0 \right)}$, and also the specific scattering matrix elements $\left\langle f | H_{int}^{(e,a)} | i \right\rangle$.

For energy/momentum transfer in electron energy loss spectrum (EELS), the wavepacket acceleration as the pointer shift is thus obtained as $\Delta E=\sum_{p,\nu} \left|c_{p,\nu}^{(0)}+c_{p,\nu}^{(1)(\mathrm{e})}+c_{p,\nu}^{(1)(\mathrm{a})}\right|^{2}\left(E_{p}-E_{0}\right),$ where the initial electron energy$E_{0}=\sum_{p,\nu} \left|c_{p,\nu}^{(0)}\right|^{2}E_{p}$. Note that the unnormalized final state $c_{p,\nu}^{(\mathrm{f})}=c_{p,\nu}^{(0)}+c_{p,\nu}^{(1)(\mathrm{e})}+c_{p,\nu}^{(1)(\mathrm{a})}$ has the photon-emitted (*e*) and photon-absorbed (*a*) contribution from electron-photon scattering processes, respectively. Here we expand together the expressions comprising$\Delta E$, then cancel the initial terms and rewrite as two separate terms: $\Delta E^{(1)}=2\sum_{p,\nu} \Re\left\{c_{p,\nu}^{(0)*}\cdot c_{p,\nu}^{(1)(\mathrm{e})}+c_{p,\nu}^{(0)*}\cdot c_{p,\nu}^{(1)(\mathrm{a})}\right\}\left(E_{p}-E_{0}\right)$ and$\Delta E^{(2)}=\sum_{p,\nu} \left|c_{p,\nu}^{(1)(\mathrm{e})}+c_{p,\nu}^{(1)(\mathrm{a})}\right|^{2}\left(E_{p}-E_{0}\right)$, where $\mathfrak{R}$ stands for the real part of the argument. The phase-independent term $\Delta E^{(2)}$ is the term that corresponds to photon emission rate, as derived from the Fermi’s Golden Rule (FGR) [10-12], while the phase-dependent term $\Delta E^{(1)}$ that originates from quantum interference between the initial state and scattered state is an additional contribution which is usually omitted in the formulation of FGR but leads to the classical linear acceleration [10].

The evolution operator is given by

The quantum recoil of the electron is found from substituting in the energy dispersion relation, expanded to second order:$E_{p}=c\sqrt{m^{2}c^{2}+p^{2}}\approx\varepsilon_{0}+\text{v}_{0}\left(p-p_{0}\right)+\frac{\left(p-p_{0}\right)^{2}}{2m^{*}}$, determined by the delta functions and $\mathrm{p}_{0}=\gamma_{0}m\text{v}_{0},m^{*}=\gamma_{0}^{3}m$. Then the first-order perturbation coefficients are explicitly given by

(B1)

with and $\overline{\theta}=\left(\frac{\omega}{\text{v}_{0}}-q_{z}\right)L$is the classical interaction ‘detuning parameter’. The normalized photon exchange coefficient is.

To derive the energy transfer $\Delta E=\sum_{p,\nu} \left|c_{p,\nu}^{(0)}+c_{p,\nu}^{(1)(\mathrm{e})}+c_{p,\nu}^{(1)(\mathrm{a})}\right|^{2}\left(E_{p}-E_{0}\right),$ the integration over *p* in (B1) should be carried out with the Gaussian distribution function of the drifted electron amplitude in momentum space: and $\tilde{\Delta}_{p}^{2}\left(t_{D}\right)=\Delta_{p_{0}}^{2}\left(1+i\xi t_{D}\right)^{-1}$. For the phase-independent energy transfer emission term$\Delta E^{(2)}=\sum_{p,\nu} \left|c_{p,\nu}^{(1)(\mathrm{e})}+c_{p,\nu}^{(1)(\mathrm{a})}\right|^{2}\left(E_{p}-E_{0}\right)$, this involves the following integration:

(B2)

and similarly, for the absorption term:

(B3)

For the phase-dependent energy transfer emission part ($\Delta E^{(1)}=2\sum_{p,\nu} \Re\left\{c_{p,\nu}^{(0)*}\cdot c_{p,\nu}^{(1)(\mathrm{e})}+c_{p,\nu}^{(0)*}\cdot c_{p,\nu}^{(1)(\mathrm{a})}\right\}\left(E_{p}-E_{0}\right)$):

(B4)

Analogously, for the absorption term:

(B5)

where we define the decay parameter

(B6)

and $\Gamma_{0}=\frac{2\pi}{\beta_{0}}\left(\frac{\Delta_{z}}{\lambda}\right)$, . Note that in all cases, we used the approximation in the last steps of calculation. Also, note that the imaginary part may contribute to an additional phase within the cosine in the case of very long drift time $t_{D}$.

1. **The classical electromagnetic field correspondence of the coherent photon state**

Within the treatment of quantum electrodynamics, the simplified interaction Hamiltonian in the Coulomb gauge ($\nabla\cdot\hat{\text{A}}=0$) is given by

$H_{I}\left(t\right)=-\left(\frac{e}{\gamma_{0}m}\right)\hat{\text{A}}\cdot\text{p}$, (C1)

where the vector potential in second quantization (box quantization with volume V) on a grating is given by (A10)

(C2)

leading to the electric field

(C3)

where $\hat{\varepsilon}_{q}$is the normalized polarization vector (this axial slow-wave field component would be one of the Floquet space harmonics of the radiation mode in the periodic structure of the Smith-Purcell interaction scheme or the axial component in a dielectric structure in Cerenkov radiation interaction schemes). For the given laser field with a longitudinal component , the corresponding coherent state $|\alpha\rangle$ for the m^th^-order Floquet harmonics is then obtained as

(C4)

where we consider one harmonic of monochromatic near-field photonic excitation with synchronization condition, $\omega/q_{zm}=v_{0}$and the laser frequency is fixed at $\omega$, and we obtain $\text{E}_{\text{c}}=|\alpha|\tilde{E}_{q}$. We should note that the quantization of near-field excitation is a non-trivial issue that relates to the specific configuration of near-field harmonics distribution and spectrum on the grating and the laser illumination condition. Here for our simplified model, we use the free-space quantization for the interaction light field and naively absorb all the structural information of the near-field into the effective dielectric constant $\varepsilon_{eff}$.

1. **The Wigner function representation of our measurement theory in phase space and its comparison with decoherence theory**

The comparison between our measurement theory and decoherence theory can be also slightly explained in Figs. 2a and 2b. Based on the conventional QED formulation, we can obtain three spectral sidebands (i.e., one initial sideband, single-photon-emitted sideband and single-photon-absorbed sideband) in the final electron wavefunction $\psi_{p}=\psi_{0}\left( p-p_{0} \right)+\psi_{-1}(p-p_{0}-\hbar\omega/v_{0})+\psi_{1}(p-p_{0}+\hbar\omega/v_{0})$. If we present the final electron wavefunction in phase space as Wigner function,

$W\left( p,z \right)=\frac{1}{\pi}\int\psi_{p+\frac{q}{2}}\psi_{p-\frac{q}{2}}^{*} e^{iqz/\hbar}dq$ (D1)

then we can explicitly obtain $W\left( p,z \right)=W_{0,0}+W_{1,1}+W_{-1,-1}+2\mathfrak{R\{}W_{0,1}+W_{0,-1}+W_{1,-1}\}$ from the first-order perturbative approximation. These interference fringes can be viewed explicitly in Fig. 2c. The interference parts between sidebands in the point-particle limit of electron wavepacket leads to the central momentum shift of the final electron Wigner function, as shown in Fig. 2a. However, when considering decoherence, all interference terms are suppressed $2\mathfrak{R}\left\{ W_{0,1}+W_{0,-1}+W_{1,-1} \right\}=0$. In this situation, the final electron momentum distribution symmetrically broadens, with no wavepacket acceleration in the spectrum, as similarly shown in Fig. 2b.

It has to be noted that Fig. 2b is the interaction between classical electron and quantum photon ($|\nu\rangle$). Thus, these interference terms do not exist intrinsically because of the orthogonality relation of the associated photon states with the electron sidebands, that is$\left\langle\nu| \nu\pm1 \right\rangle=0$. The natural suppression of quantum interference in the cases 2b and 2d is the same as the prediction from the decoherence program, in which the photon state acts like the environment state.

1. **The projective-to-weak measurement transition of periodically bunched electron beams: the net linear energy transfer of the PINEM spectrum**

Here, we will demonstrate the weak measurement of an electromagnetic field with a periodically bunched electron having PINEM photon sidebands (with discrete spectral spacing $\hbar\omega$). In the context of quantum measurement theory, the measuring apparatus is a train of periodically bunched attosecond electron pulses. Considering the free electron laser interaction as having a short duration (neglecting the chirp during the interaction), we can describe the interaction in the following simplified Hamiltonian,

$H=H_{0}+H_{int},$ (E1)

where the first term is the free electron Hamiltonian (A8), and the second term is the interaction part (C1). We ignore the dynamics of the optical field during the short interaction time. Based on the Schrödinger equation, we shall describe the interaction of an electron with two sequential near-fields in the following mathematical form,

$\left| \left. \psi_{f} \right\rangle=A\left( g_{2} \right)U\left( t_{D} \right)A\left( g_{1} \right) \right|\left. \psi_{i} \right\rangle,$ (E2)

where $\psi_{i,f}$ are the initial unperturbed and finial modulated free electron wave functions. The evolution operator is divided into three parts with $A\left( g_{1} \right)$ being the interaction on the first modulator, $U\left( t_{D} \right)$ being the free drift between two near-field interactions, and $A\left( g_{2} \right)$ being the interaction on the second analyzer. Without loss of generality, the three operators can be modeled as

$\begin{matrix} A\left( g_{1,2} \right)=\exp\left( -2i\left| g_{1,2} \right|\sin\left( \frac{\omega z}{v_{0}}+\phi_{1,2} \right) \right), \\ U\left( t_{D} \right)=\exp\left( -iH_{0}\left( \frac{\partial}{\partial z} \right)t_{D} \right). \end{matrix}$ (E3)

with $g_{1,2}=\frac{e}{2\hbar\omega}\int F\left( z \right)e^{-i\omega z/v_{0}}dz=2\sqrt{\nu_{0}} \tilde{\Upsilon}$ as we derived previously. The longitudinal near-field $F\left( z \right)$ can be described by an effective traveling sinusoidal function [Hommelhoff-PRL-2019, Ropers-NPhoton-2017] (omitting the transverse coordinate). The coupling constants $g_{1,2}$ are complex, and the relative phase difference $\left| \phi_{1}-\phi_{2} \right|$ is the time delay (delay 2 in Fig. 4b) between the two interactions. There is a relevant time delay (delay 1 in Fig. 4b) between the initial wave function and the modulator near-field given by $\left| \phi_{1}-Arg(\psi_{i}) \right|$. The free electron Hamiltonian in the nonrelativistic limit is given by Eq. A8, where only the longitudinal derivative is involved $\nabla\to\frac{\partial}{\partial z}$.

In principle. the specific electron-photon interaction process as described by (E2) can be solved analytically. In our case, we are curious about obtaining the final accelerated electron wave function numerically with an asymmetric PINEM spectrum. Let us assume the initial electron wave function to be a chirped Gaussian (Eq. 1), given by

$\psi_{i}\left( z, t \right)=\frac{e^{\frac{i\left( p_{0}z-\varepsilon_{0}t \right)}{\hbar}}}{\left( 2\pi\Delta_{z_{0}}^{2} \right)^{\frac{1}{4}}\sqrt{1+i\xi t}}\exp\left( \frac{\left( z-v_{0}t \right)^{2}}{4\Delta_{z_{0}}^{2}\left( 1+i\xi t \right)} \right),$ (E4)

with the initial wave packet waist $\Delta_{z_{0}}=\hbar/2\Delta_{p_{0}}$. For the condition that $\Delta_{z_{0}}\gg\beta\lambda$ corresponds to the quantum plane-wave limit (see Fig. 3d), the spectrum of modulated intermediate electron state ($A\left( g_{1} \right)\left. \psi_{i} \right\rangle$) will produce symmetric photon spectral sidebands. The free drift process would translate the imprinted energy modulation into a periodic charge density modulation, yielding the attosecond bunched electron pulses in the propagated intermediate state ($U\left( t_{D} \right)A\left( g_{1} \right)\left. \psi_{i} \right\rangle$ (see the inset of Fig. 4b). The attobunched electron pulse size is much smaller than the wavelength. The periodicity of the pulse trains is synchronized with the optical cycle so that the whole pulse train can be accelerated in a classical fashion. It is a surprising feature because the net energy transfer in the second interaction of the analyzer ($|\left. \psi_{f} \right\rangle$) emerges from the quantum PINEM interaction by exchanging discrete photon quanta.

Fig. S1 demonstrates the density modulation and corresponding energy modulation of the final free electron wave function. Fig. S1a-d shows the final modulated electron after interacting with the modulator near-field without and with free drift, respectively. With the control of the drift duration ($t_{D}$), we can produce the optimal charge density bunching (see Fig. S1c). Note that the drifted PINEM spectrum (S1d) is the same as the undrifted case (S1b) because the momentum is a good quantum number in the duration of free propagation. Fig. S1e-h shows the final modulated electron after passaging the interaction region of the analyzer. Figs. S1e and S1f are calibration scenarios where the two interaction regions are too close, and no intermediate free drift occurs. We can easily observe a broader PINEM spectrum in Fig. S1f. The most striking result is the accelerated PINEM spectrum for the periodically bunched electron wave function, as shown in Fig. S1h. The whole energy shift in PINEM stems from the quantum interference between photon sidebands. From a classical perspective, each attobunch experiences the dynamics of point-particle in the presence of a synchronized electromagnetic field. The asymmetric spectral feature validates the possibility of performing a weak measurement of the analyzer near-field via the bunched and synchronized electron pulse trains instead of a single bunched electron.

To assess the classical emergence of weak measurement in the analyzer interaction regime, Fig. S2 demonstrates the linear net acceleration of the periodically bunched pulses trains. Fig. S2a shows that the optimal drift duration is dependent on the modulator near-field strength ($g_{1}$). For a fixed drift duration (corresponding to the distance between the modulator and the analyzer), the energy gain is maximal at the optimal bunched case, whereas the under bunched and over bunched electron beams cannot be accelerated efficiently. Fig. S2b shows the striking linear dependence of the energy gain as a function of the analyzer near-field strength ($g_{2}$), even for the under bunched electron pulse trains. The quantum analysis (Fig. S2) is in nicely consistent with the experimental data (Fig. 4d in the main text). The relevant parameters are given in the figures.

Figure S3 depicts how bunching the electron in time can shrink the electron wavefunction's size beyond the condition of the quantum-to-classical transition for Gaussian electron pointers. The crucial result we found beyond the limit of quantum-to-classical measurement transition is refers to the bunched electron, $2\Delta_{b}<T<2\Delta_{t}$. Notice that $2\Delta_{b}$ is the micro bunch size, and $2\Delta_{t}$ is the initial unbunched electron size. We cannot accelerate the whole electron wavefunction due to $T<2\Delta_{t}$. However, we can accelerate each bunched electron pulse because the bunch size is smaller than the optical cycle, $2\Delta_{b}<T$. On the other hand, to ensure all the bunches are net accelerated, we require the bunching period to be the same as the optical cycle (or multiple an integer number of the optical cycle). In this sense, even though the whole electron wavefunction is wider than the light wavelength, we still may be able to achieve a net acceleration (see Fig. S2).


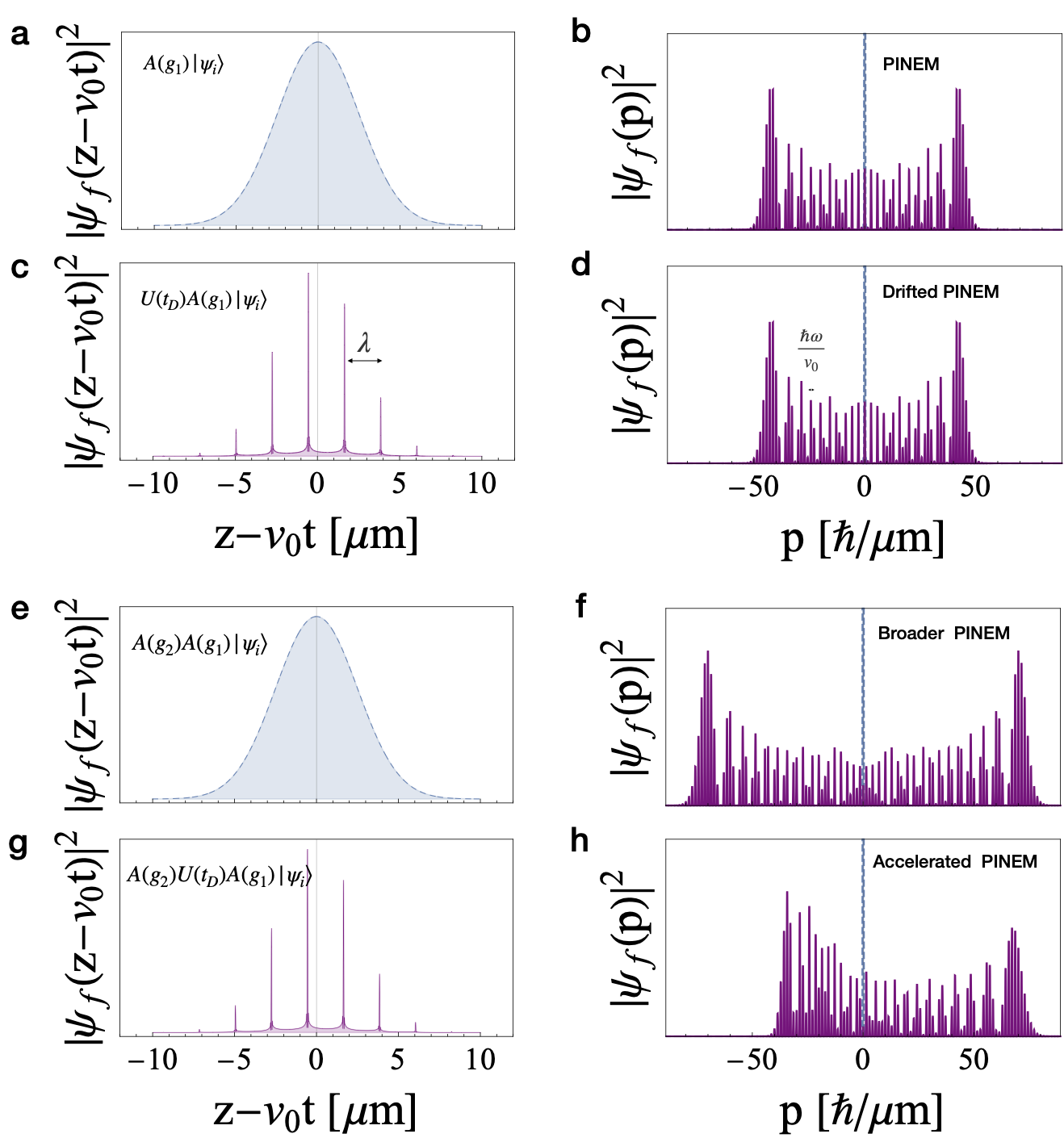


Fig. S1: The final density modulation and energy modulation of free electron wave function in free-electron and photon interaction. (a, b) The modulated density and energy distribution after passing the modulator interaction region ($\boldsymbol{A}\left( \boldsymbol{g}_{\boldsymbol{1}} \right)\left. \boldsymbol{\psi}_{\boldsymbol{i}} \right\rangle$). (c, d) The modulated density and energy distribution after free drift duration ($\boldsymbol{U}\left( \boldsymbol{t}_{\boldsymbol{D}} \right)\boldsymbol{A}\left( \boldsymbol{g}_{\boldsymbol{1}} \right)\left. \boldsymbol{\psi}_{\boldsymbol{i}} \right\rangle$). (e, f) The modulated density and energy distribution after passing the analyzer interaction region ($\boldsymbol{A}\left( \boldsymbol{g}_{\boldsymbol{2}} \right)\boldsymbol{A}\left( \boldsymbol{g}_{\boldsymbol{1}} \right)\boldsymbol{|}\left. \boldsymbol{\psi}_{\boldsymbol{i}} \right\rangle$), but without considering the free drift. (g, h) The modulated density and energy distribution after passing the analyzer interaction region ($\boldsymbol{A}\left( \boldsymbol{g}_{\boldsymbol{2}} \right)\boldsymbol{U}\left( \boldsymbol{t}_{\boldsymbol{D}} \right)\boldsymbol{A}\left( \boldsymbol{g}_{\boldsymbol{1}} \right)\boldsymbol{|}\left. \boldsymbol{\psi}_{\boldsymbol{i}} \right\rangle$).


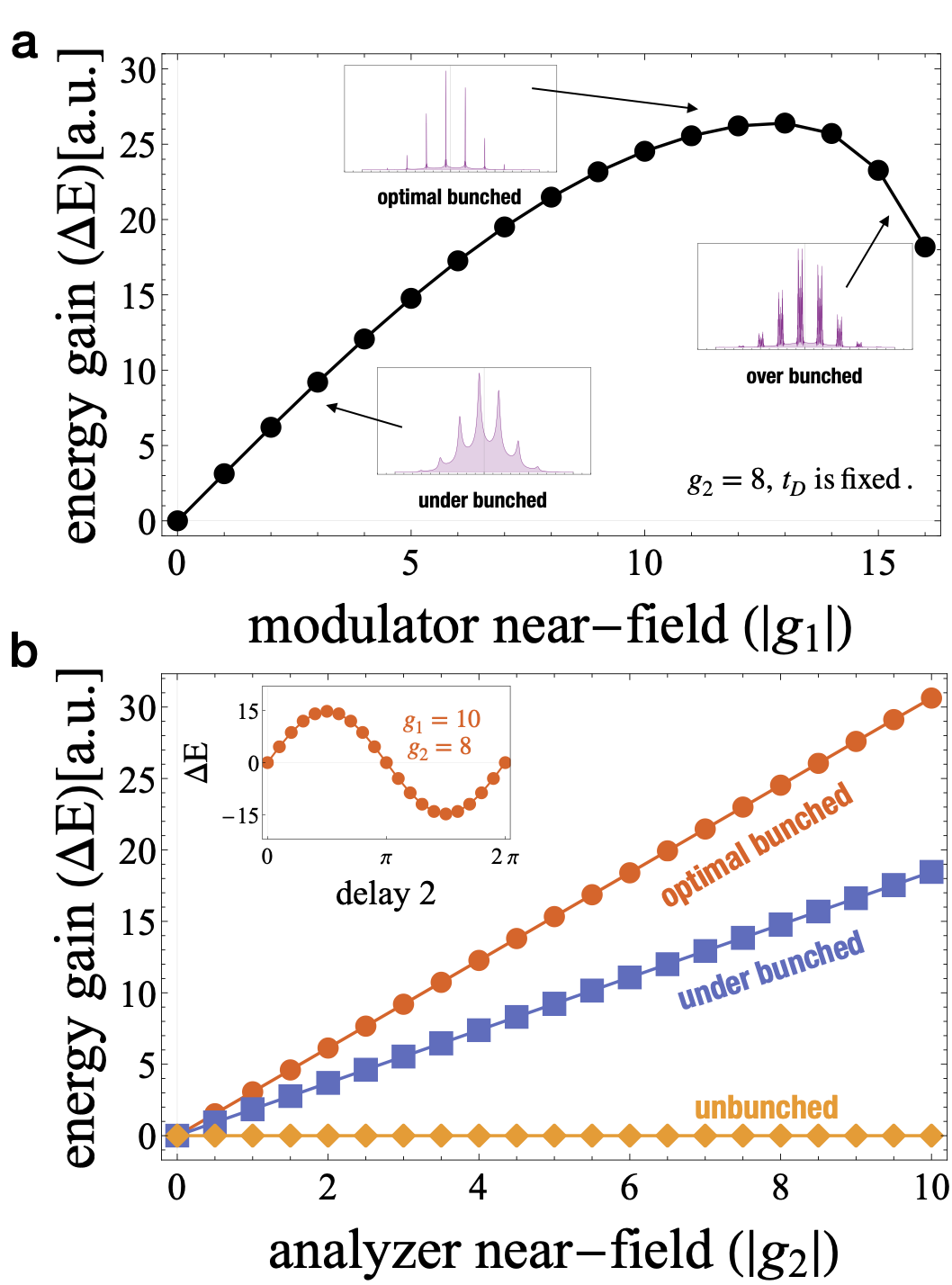


**Fig. S2:** **The linear net acceleration of periodically bunched electron pulses trains showing the weak measurement of the analyzer near-field.** (a) Numerical simulations of the energy gain of the electron pulses trains having traversed both modulator and analyser stage, as a function of the modulator near-field strength. The inset shows the bunching state at the analyser. The resulting net energy gain is plotted here, for under-bunched, optimally bunched, and over-bunched electron pulse trains. The analyser field strength was kept constant here. (b) The opposite situation: Here, the modulator field strength is kept constant at three different values, indicated by the colors: For an unbunched beam, clearly no net energy gain is expected, while it becomes maximal for the optimally bunched beam (orange). The linear dependence of net acceleration for the bunched electron pulses as a function of the analyzer near-field strength is the main result here, as also seen in the experiment. The inset shows the phase dependence of the point-particle-like acceleration of periodic bunches.


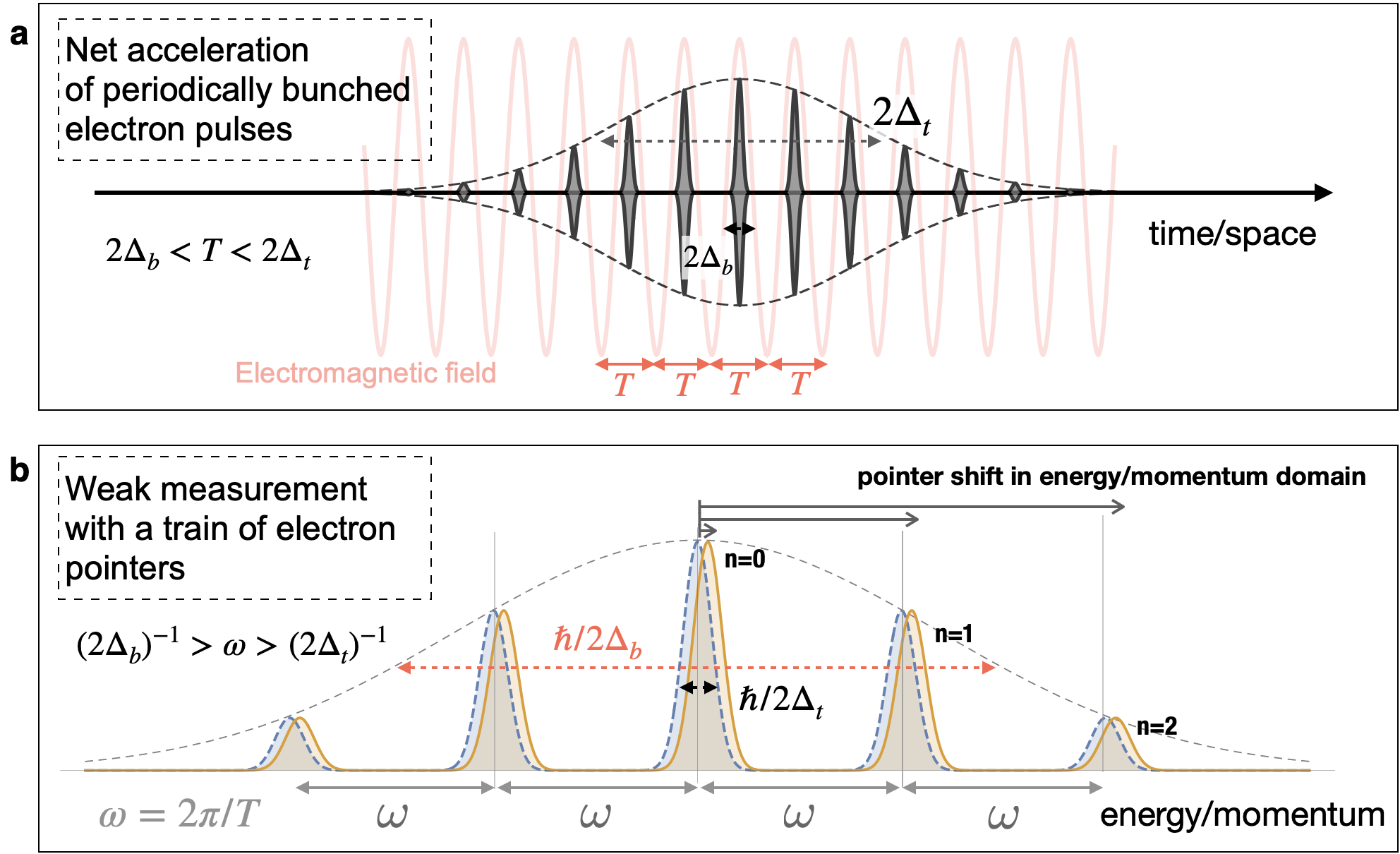


**Fig. S3: The periodic spectral structures of the measuring electron pointer can exceed the limit of the quantum-to-classical measurement transition.** As a result, we can obtain a net acceleration of the PINEM electrons in the region where $2\Delta_{b}<T<2\Delta_{t}$. In the time/space domain (a), these PINEM electrons behave like periodically bunched pulses, while in the energy/momentum domain (b), they have a spectrum dressed by multiphoton sidebands. This figure can explain the net energy transfer of the pre-bunched electrons in the DLA setup (see Fig. 4b).


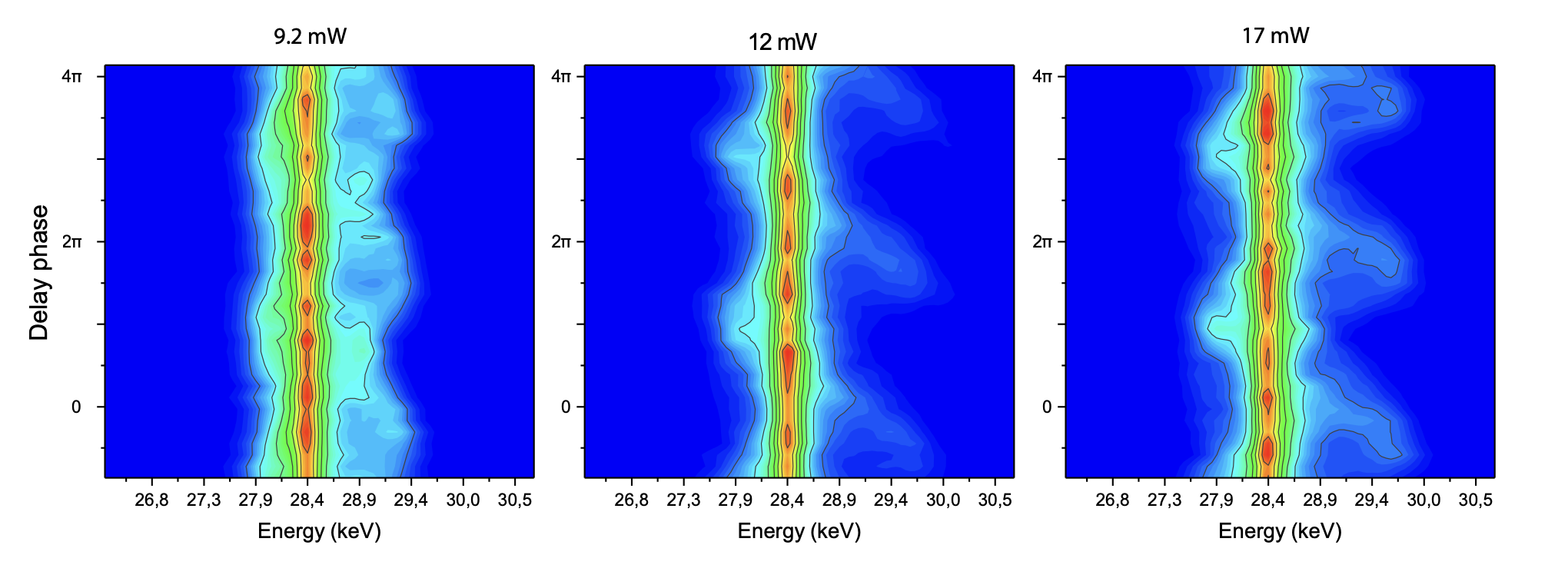


**Fig. S4:** **Three example spectrograms showing the energy distribution as function of relative phase between laser pulses driving the modulator and the analyser.** The delay axis is presented in units of the optical cycle (6.45fs). Three different modulator powers of 9.2 mW, 12 mW, and 17mW are shown, corresponding to peak field strengths of (141, 161, 191) MV/m. The colour shows electron counts, normalized to the maximum for each data set individually. The "acceleration peak" in Fig. 4 of the main text is found from slices of these data. The "5% maximum" is the maximum acceleration found where 5% of the maximum count rate shows the largest energy gain.
